# Spatial Size Can Affect Social Categorization of the Rich and the Poor

**DOI:** 10.3389/fpsyg.2020.01914

**Published:** 2020-08-11

**Authors:** Xiaobin Zhang, Zhe Zhang

**Affiliations:** ^1^School of Psychology, Shaanxi Normal University, Xi’an, China; ^2^School of Psychology, Northwest Normal University, Lanzhou, China

**Keywords:** perceptual simulation, social categorization, the rich, the poor, spatial size

## Abstract

Previous research has shown that representation of certain social-category knowledge, such as that regarding gender, involves the process of perceptual simulation. The present research extended these findings and explored whether social categorization based on wealth, which is an important dimension of social categorization, involved perceptual simulation of spatial size. In Experiment 1, we used high- and low-income occupations as stimuli; categorization of high-income occupations presented in larger font was faster relative to that of those presented in small font, and vice versa for low-income occupations. In Experiments 2, 3, and 4, we used high-income occupations without social power and low-income occupations, names designated as those of rich and poor people, and idioms describing wealth and poverty as stimuli, respectively. All three experiments showed that responses to wealth-related stimuli in larger font were faster relative to those to the same stimuli in small font, and vice versa for poverty-related stimuli. These results suggest that social categorization based on wealth is grounded in perceptual simulation of spatial size.

## Introduction

Models of grounded cognition assume that cognitive representations of concepts include sensory, motor, and introspective states, which are activated via simulations (Barsalou, 1999, 2008). Recently, researchers found that a fundamental social-cognitive process, social categorization, was represented by sensorimotor activity, and cognitive representations of social categories, such as gender and political persuasion, were grounded in perceptual simulation and sensorimotor activity (Slepian et al., 2011, 2012; Zhang et al., 2014; Slepian, 2015). Extending this literature, the current work examined whether cognitive representations of social categories of the rich and the poor, which is one of most important social categorization dimensions (Leahy, 1981; Harvey and Bourhis, 2013), involved perceptual simulation of the spatial size dimension. Specifically, we explored the possibility that spatial size perceptual simulation shaped the categorization of the rich and the poor. How abstract concepts are mentally represented is an essential question within the domain of cognitive psychology. A wide array of research found that human cognition is grounded in and shaped by sensorimotor experiences, and that our conceptual representations are recruited into partial simulations, which reenact various embodied states (Barsalou, 2008). Exploring the connection between the social categories of the rich and the poor and the perceptual simulation of the spatial size dimension is an important research issue on embodied cognition and social cognition (Cloutier et al., 2005; Macrae et al., 2005; Slepian et al., 2011). First, it can extend the representation of social-category knowledge involving the process of perceptual simulation to wealth. Second, social categorization is the basis and prerequisite for stereotype activation, stereotype application, and even prejudice and discrimination (Allport, 1954), and examining the effect of spatial size on the social categorization of the rich and the poor may provide a new perspective for reducing stereotypes, prejudices, and discrimination against the rich and the poor.

Slepian et al. (2011) were the first to find that the process of social categorization involving gender was embodied within sensorimotor experience. They showed that perceivers’ gender-category representations included the sensorimotor experience of different levels of hardness, with masculinity associated with toughness and femininity associated with tenderness. Specifically, participants continuously squeezed a soft or hard ball while categorizing sexually ambiguous faces, and the results indicated that faces were categorized as feminine by participants squeezing the soft ball more often relative to those squeezing the hard ball (Slepian et al., 2011). Inspired by this research, Zhang et al. (2014) found that the process of gender categorization involved perceptual simulation of spatial height and size dimensions. Their results showed that responses to masculine faces were faster when they were in the higher position, rather than the lower position, and vice versa for feminine face categorization. Further, categorization of men’s names depicted in larger font was faster relative to that of men’s names depicted in smaller font, whereas opposite response patterns were observed for women’s names (Zhang et al., 2014). Consistent with the findings reported by Zhang et al. (2014). Lamer et al. (2017) suggested that spatial position affected visual perception of gender, in that participants perceived greater femininity in faces that appeared lower in space. In addition to the representation of knowledge regarding gender social categories, Slepian (2015) found that the categorization of Republicans and Democrats involved sensorimotor processes. Specifically, half of the faces in their study were presented next to a hard object (e.g., a rock), and the remaining faces were presented next to a soft object (e.g., a pillow); participants categorized the target faces as Republicans or Democrats. The results indicated that both perceptual simulation metaphors and sensorimotor processes (toughness) could bias social categorization of political persuasion, with Republicans associated with sensorimotor experiences of hardness and Democrats associated with sensorimotor experiences of softness.

The categorization of individuals according to wealth is another very important and common dimension of social categorization, and social stratification based on wealth is obvious in most countries (Leahy, 1981; Harvey and Bourhis, 2013). For example, children aged 3–5 years have been seen to understand the terms *poor* and *rich* and classify people as such (Ramsey, 1991), and those aged 6–11 years begin to believe that poor and rich people possess differences in certain traits such as intelligence and effort (Leahy, 1981). One of the largest perceptual differences between rich and poor involves differences in spatial size. For example, in China, because rich people are able to eat nutritious food and do not need to engage in high-intensity manual labor, they are often perceived as more likely to be heavier relative to poor people. In fact, Chinese researchers have found that the weight and height of adolescents in the wealthy areas of China is significantly greater than those of adolescents in the poor areas of China (Han and Xu, 2014). In addition, in everyday life, being rich is associated with the possession of larger houses and vehicles. We often describe wealth using words related to large spaces, such as in the idiom “rich family and large room (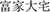).” In contrast, we often describe poverty using words related to small spaces, such as in the Chinese idiom “too poor to have place to lay a cone (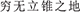).” Therefore, perceptual simulations of spatial size can provide a foundation for representations of wealth and poverty, and perceivers can call the bipolar extremes of this difference to mind when they think of the rich (“bigger spatial size”) and the poor (“small spatial size”).

It should be pointed out that previous research has examined spatial size in terms of social power, which, although distinct from wealth, can provide ideas of how spatial size may relate to wealth. These studies showed that categorization of groups with different levels of power, based on the process of power judgment, involves vertical position and the perceptual simulation of spatial size (Schubert, 2005; Schubert et al., 2009). For instance, Schubert (2005) found that judgment of a group’s power was influenced by the group’s vertical position in space, and reactions to power were faster when it was presented at the top, relative to the bottom of the screen. Further, Schubert et al. (2009) showed that power categorization was affected by spatial size, and reaction times to stimuli representing the categorization of power presented in larger font were faster relative to those depicted in smaller font, whereas opposite response patterns were observed for stimuli representing powerlessness. Although this research indicated that groups with high and low social power were associated with perceptual simulations of vertical position and spatial size, the findings only indicated that sensorimotor processes could influence the process of power judgment rather than the process of social categorization. In addition, social power and wealth are not equivalent concepts. Social power means the ability to influence others or control others’ outcomes (Fiske, 1993). Wealth, on the other hand, is the possession of a large amount of money. Although some people with social power are wealthy, individuals with power are not necessarily wealthy, and wealthy people do not necessarily possess social power.

We proposed that the social categorization process based on wealth, which is an important social categorization dimension (Leahy, 1981; Harvey and Bourhis, 2013), may involve sensory metaphors of different spatial sizes, and the aim of the current study was to determine whether perceptual simulation of spatial size would affect the categorization of the rich and the poor. We hypothesized that categorization as rich would be faster when stimuli representing rich people were presented in large spatial size (vs. small spatial size), and categorization as poor would be faster when stimuli representing poor people were presented in small spatial size (vs. large spatial size). In Experiment 1, we used high-income and low-income occupations to represent wealth and poverty, respectively (generally speaking, in China, high-income earners have more wealth than low-income earners), and explored whether spatial size affected the categorization of these occupations. In Experiment 2, we compared high-income occupations without social power and low-income occupations and examined whether spatial size affected the categorization of high- and low-income occupations. In Experiment 3, we explored whether spatial size affected categorization of names designated as those of rich and poor people. In Experiment 4, we explored whether spatial size affected categorization of abstract idioms describing wealth and poverty.

## Experiment 1

### Materials and Methods

#### Ethics Statement

This study was reviewed and approved by the committee for the protection of subjects at Northwest Normal University, School of Psychology Ethics Committee. Written consent was obtained from all participants before the experiment, according to the established guidelines of the committee. This procedure was followed in all experiments.

#### Participants and Design

Thirty Chinese university students (*M* = 21.27, *SD* = 2.79 years; six men) were recruited as participants. Using G^∗^Power 3.1 (Faul et al., 2009), we calculated an optimal sample size of *N* = 29 using a conventional threshold of α = 0.05 for Type I error, a power of 0.8, and an effect size of η*_p_*^2^ = 0.07. Based on the sample size in a similar previous study (Meier and Robinson, 2004), we decided to recruit 30 participants prior to data collection. This method of participant selection was performed for all four experiments. This experiment involved a 2 (occupation type: high income, low income) × 2 (font size: large, small) repeated measures design.

#### Materials

The experimental materials were 16 high-income and 16 low-income occupation names selected as follows. First, 78 common occupation names (such as doctor, farmer) were selected via an Internet search. Second, 15 participants who did not participate in the main experiment rated the income level of each occupation (such as *doctor*) on a five-point scale, in which “1” indicated “very low income” and “5” indicated “very high income.” These participants also rated the familiarity of each occupation on a five-point scale, in which “1” indicated “low familiarity” and “5” indicated “high familiarity.” Third, based on scores for the income dimension, 16 high-income occupations (*M* = 4.22, *SD* = 0.30) and 16 low-income occupations (*M* = 2.17, *SD* = 0.24) were selected, and a significant difference was found in the average scores of income between these selected 16 high-income and 16 low-income occupations, *t*(30) = 19.92, *p* < 0.001. The scores for familiarity for all occupations were > 3, and there was no significant difference in the familiarity scores between high- (*M* = 3.82, *SD* = 0.26) and low-income occupations (*M* = 3.71, *SD* = 0.19), *t*(30) = 1.23, *p* = 0.238.

#### Procedure

Participants were asked to sit in front of a computer screen to complete the experiment. In each trial, a fixation cross was presented at the center of the screen for 800 ms, followed by a high- or low-income occupation name in large (font size 50) or small (font size 30) font for 2,000 ms or until the participant responded. Participants were required to classify occupations according to their income (high vs. low) as quickly and as accurately as possible by pressing the “high income” or “low income” key (Meier and Robinson, 2004). The keys were counterbalanced across the participants. If the response time exceeded 750 ms, the words “Please respond quickly” appeared in red font at the center of the screen for 700 ms.

The experiment included 128 trials. Each occupation was presented twice in both large and small font respectively. Prior to the main experiment, the participants completed 24 practice trials.

### Results and Discussion

**FIGURE 1:**
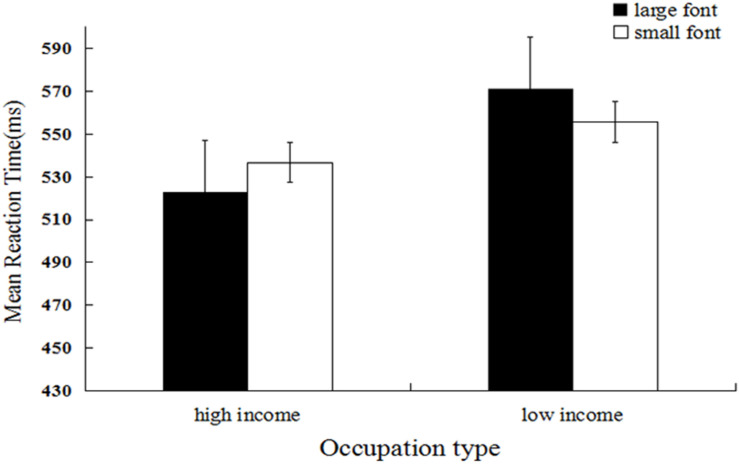
Mean reaction latency as a function of occupation type and occupation font size (Experiment 1).

Mean categorization latency was the dependent measure of interest^1^. We firstly performed log transformation of the data, however, untransformed means are reported for ease of interpretation. Response times that were outside of 3 *SD*s from the mean (32 trials, 0.83%) and trials during which errors were made (275 trials, 7.16%) were excluded from the analysis (307 trials, 7.99%). A 2 (occupation type) × 2 (font size) repeated measures ANOVA was performed to examine the participants’ reaction times. The results showed that the main effect of occupation type was significant, and the mean reaction time for high-income occupations (*M* = 530.80, *SD* = 82.33) was significantly shorter relative to that for low-income occupations (*M* = 563.24, *SD* = 101.96), *F*(1, 29) = 96.87, *p* < 0.001, η*_p_*^2^ = *0.109*. The main effect of font size was non-significant, *F*(1, 29) = 0.243, *p* = 0.622, η*_p_*^2^ = 0.001. As predicted, the interaction between occupation type and font size was significant, *F*(1, 29) = 35.19, *p* < *0.001*, η*_p_*^2^ = 0.042 (see Figure 1). Further simple effect analysis showed that the mean response time for high-income occupations in large font size (*M* = 523.04, *SD* = 82.44) was significantly shorter relative to that for those in small font size (*M* = 536.74, *SD* = 81.52), *F*(1, 29) = 23.05, *p* < 0.001, η*_p_*^2^ = 0.028. In contrast, the mean response time for low-income occupations in large font size (*M* = 571.14, *SD* = 108.43) was significantly longer relative to that for small font size (*M* = 555.75, *SD* = 94.88), *F*(1, 29) = 12.99, *p* < 0.001, η*_p_*^2^ = 0.016.

These results provided initial support for our hypothesis that the process of social categorization based on wealth involves perceptual simulation of spatial size. Wealth was associated with large spatial size, and poverty was associated with small spatial size. If a social category was consistent with the perceptual simulation (e.g., high-income occupations presented in large font), the process of social categorization based on wealth was facilitated. If a social category was incongruent with the perceptual simulation (e.g., high-income occupations presented in small font), the process of social categorization based on wealth was restricted. In Experiment 1, we used high-income occupations with social power (e.g., mayor, commander in chief, and secretary of state). Previous research has shown that the categorization of high and low levels of social power is influenced by spatial size metaphors (Schubert et al., 2009). Were the results of Experiment 1 induced by social power attached to high-income occupations? To answer this question and verify the results of Experiment 1, we examined high-income occupations without social power (e.g., doctor) in Experiment 2.

## Experiment 2

### Materials and Methods

#### Participants and Design

Thirty Chinese university students (*M* = 21.47, *SD* = 2.53 years; six men) were recruited as participants. This experiment involved a 2 (occupation type: high income without social power, low income) × 2 (font size: large, small) repeated measures design.

#### Materials

The experimental materials were 16 high-income occupations without social power (e.g., doctor, college teacher, host, and bank staff) and 16 low-income occupations (the same as occupations used in Experiment 1). The selection of high- and low-income occupations occurred as follows. Initially, 78 common occupations were selected based on an Internet search. Nineteen subjects who did not participate in the main experiment used a five-point scale to rate income (low = 1, high = 5) and familiarity (unfamiliar = 1, familiar = 5). Based on scores for the income dimension, we chose 16 high-income occupations without social power (*M* = 4.21, *SD* = 0.38) and 16 low-income occupations (*M* = 2.17, *SD* = 0.24), and the average scores of income for the 16 high-income occupations were significantly higher relative to those for the 16 low-income occupations, *t*(30) = 20.47, *p* < 0.001. The familiarity scores for the 32 occupations were > 3, and there was no significant difference between the familiarity scores for high-(*M* = 3.75, *SD* = 0.22)and low-income occupations (*M* = 3.71, *SD* = 0.19), *t*(30) = 0.49, *p* = 0.63.

We also asked another 15 participants to judge the social power of high-income occupations used in Experiments 1 and 2 (occupations without social power). Participants rated the social power of each occupation (such as doctor and president) on a nine-point scale, in which “1” indicated “very low social power” and “9” indicated “very high social power.” As we expected, the mean scores on the dimension of social power of the 16 occupations (*M* = 2.36, *SD* = 0.45) used in Experiment 2 is lower than that of the occupations (*M* = 7.73, *SD* = 0.41) used in Experiment 1, *t*(30) = 34.92, *p* < 0.001.

#### Procedure

The procedure was the same as that used in Experiment 1. The experiment included 128 trials. Each occupation was presented twice in both large and small font respectively.

### Results

**FIGURE 2:**
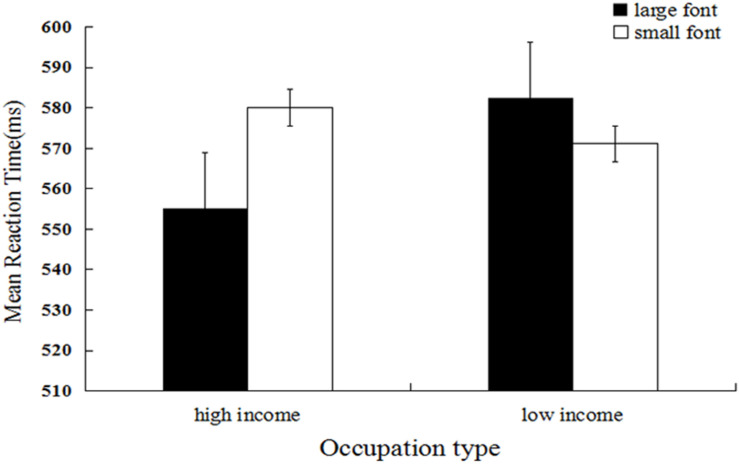
Mean reaction latency as a function of occupation type (low-income occupation and high-income occupation without social power) and occupation font size (Experiment 2).

Mean categorization latency was the dependent measure of interest^2^. We firstly performed log transformation of the data, and the untransformed means are reported. Response times that were outside 3 *SD*s from the mean (27 trials, 0.70%), and trials during which errors were made (415 trials, 10.8%) were excluded from the analysis (442 trials, 11.51%). A 2 (occupation type) × 2 (font size) repeated measures ANOVA was performed. The results showed that the main effect of occupation type was significant, and the mean reaction time for high-income occupations (*M* = 567.23, *SD* = 104.45) was significantly shorter relative to that for low-income occupations (*M* = 576.71, *SD* = 116.93), *F*(1, 29) = 17.01, *p* < 0.001, η*_p_*^2^ = *0.024*. The main effect of font size was non-significant, *F*(1, 29) = 3.05, *p* = 0.081, η*_p_*^2^ = 0.004. As expected, the interaction between occupation type and font size was significant, *F*(1, 29) = 22.11, *p* < 0.001, η*_p_*^2^ = 0.031 (see Figure 2). Further simple effect analysis showed that the mean response time for high-income occupations in large font size (*M* = 555.20, *SD* = 101.42) was significantly shorter relative to that for small font size (*M* = 580.03, *SD* = 106.15), *F*(1, 29) = 20.17, *p* < 0.001, η*_p_*^2^ = 0.028. In contrast, the mean response time for low-income occupations in large font size (*M* = 582.52, *SD* = 117.09) was significantly longer relative to that for low-income occupations in small font size (*M* = 571.12, *SD* = 116.58), *F*(1, 29) = 4.26, *p* = 0.039, η*_p_*^2^ = 0.006.

Using high-income occupations without social power, the results of Experiment 2 replicated the findings of Experiment 1, demonstrating that social categorization based on wealth involved the perceptual simulation of spatial size. It should be pointed out that high-income occupations may indicate other information, such as prestige, which may affect the effect found in Experiments 1 and 2. In order to prove the association between spatial size and the rich and the poor more broadly, rather than with occupation specifically, we used names designated as those of rich and poor people in Experiment 3. This was done to ensure the validity of the first two experiments. In Experiment 4, we used more abstract idioms describing wealth and poverty to further verify the results found in Experiments 1 and 2.

## Experiment 3

### Materials and Methods

#### Participants and Design

Thirty Chinese university students (*M* = 21.46, *SD* = 2.22 years; seven men) were recruited as participants. This experiment involved a 2 (name type: rich, poor) × 2 (font size: large, small) repeated measures design.

#### Materials

Thirty Chinese full names (15 men’s names and 15 women’s names) used in a previous study were selected (Zhang et al., 2014). All the names consisted of three characters, such as “
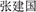
” and “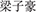,” and were judged as commonly used names by 28 participants who did not participant in the main experiment (participants judged whether the names are commonly used by indicating either a “yes” or a “no,” and all the names were judged as being commonly used by all the participants).

#### Procedure

Participants were asked to sit in front of a computer screen. They were informed that names in red or blue font would appear successively on the screen and that the names in red (blue) font were those of rich individuals, while those in blue (red) font were of poor individuals. The match between colors (red/blue) and rich/poor was counterbalanced between participants.

The task was to determine whether each name was that of a poor or rich person and press the corresponding “the poor” or “the rich” key on a keyboard (Meier and Robinson, 2004). In each trial, a fixation cross was presented at the center of the screen for 800 ms, followed by a name in red or blue font for 2,000 ms or until the participant responded. Participants were required to respond as quickly and accurately as possible. Before the formal experiment, participants completed 20 practice trials, and an accuracy rate of 95% was required. If the correct rate did not reach 95%, the participants needed to practice again until the accuracy rate reached 95%. There were 120 trials in the experiment. Each name was presented twice in both large (50) and small (30) font respectively.

### Results and Discussion

**FIGURE 3:**
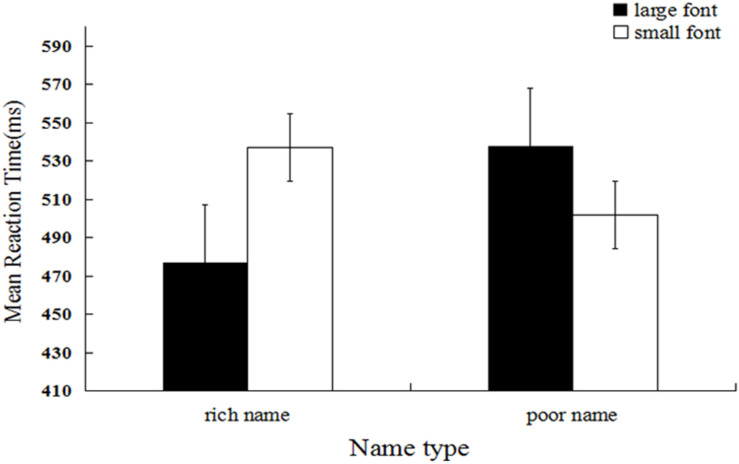
Mean reaction latency as a function of name type and name font size (Experiment 3).

Mean categorization latency was the dependent measure of interest^3^. We firstly performed log transformation of the data, and the untransformed means are reported. Response times that were outside 3 *SD*s from the mean (1 trial, 0.03%) and trials during which errors were made (177 trials, 4.92%) were excluded from the analysis (178 trials, 4.94%). A 2 (name type) × 2 (font size) repeated measures ANOVA was performed. The results showed that the main effect of name type was significant, and the mean reaction time for rich names (*M* = 506.36, *SD* = 129.01) was significantly shorter relative to that for poor names (*M* = 519.45, *SD* = 127.83), *F*(1, 29) = 20.68, *p* < 0.001, η*_p_*^2^ = 0.025. The main effect of font size was significant, and the mean reaction time for large size (*M* = 506.76, *SD* = 125.99) was significantly shorter relative to that for small size (*M* = 519.09, *SD* = 130.86), *F*(1, 29) = 21.47, *p* < 0.001, η*_p_*^2^ = 0.026. As expected, the interaction between name type and font size was significant, *F*(1, 29) = 186.77, *p* < *0.001*, η*_p_*^2^ = 0.186 (see Figure 3). Further simple effect analysis showed that the mean response time for names designated as those of rich people presented in large font (*M* = 476.84, *SD* = 105.83) was significantly shorter relative to that for names designated as those of poor people presented in large font (*M* = 537.07, *SD* = 143.10), *F*(1, 29) = 168.18, *p* < 0.001, η*_p_*^2^ = 0.171. In contrast, the mean response time for names designated as those of poor people presented in large font (*M* = 537.65, *SD* = 137.25) was significantly longer relative to that for names designated as those of poor people presented in small font (*M* = 501.72, *SD* = 115.27), *F*(1, 29) = 65.492, *p* < 0.001, η*_p_*^2^ = 0.074.

The results of Experiment 3 replicate the patterns in Experiments 1 and 2 but show that the effect is not specific to occupations and therefore appears to apply to wealth more broadly. As a final test of the robustness of the effect, we tested idioms describing wealth and poverty in Experiment 4.

## Experiment 4

### Materials and Methods

#### Participants and Design

Thirty Chinese university students (*M* = 24.07, *SD* = 2.16 years; 15 men) were recruited as participants. This experiment involved a 2 (idiom type: idiom describing wealth, idiom describing poverty) × 2 (font size: large, small) repeated measures design.

#### Materials

The materials were 15 idioms describing wealth and 15 idioms describing poverty. The selection process for the idioms was as follows. Initially, 53 common idioms describing poverty and wealth were selected. Fifteen college students who did not participate in the formal experiment evaluated these idioms on two dimensions: describing wealth or poverty (1 = rich, 2 = poor) and familiarity (1 = low familiarity, 5 = high familiarity). Fifteen idioms describing wealth and 15 idioms describing poverty were selected, and there was a significant difference in scores between idioms describing wealth (*M* = 1.02, *SD* = 0.03) and poverty (*M* = 2.00, *SD* = 0.00), *t*(28) = -188.62, *p* < 0.001. The familiarity scores for all idioms were > 3, and there was no significant difference in the familiarity scores between idioms describing wealth(*M* = 3.88, *SD* = 0.26)and those describing poverty (*M* = 3.98, *SD* = 0.27), *t*(28) = -1.21, *p* = 0.25.

#### Procedure

The procedure was the same as that used in Experiment 1, except that occupation names were replaced by idioms describing wealth and poverty. The experiment included 120 trials. Each idiom was presented twice in both large and small font respectively.

### Results and Discussion

**FIGURE 4:**
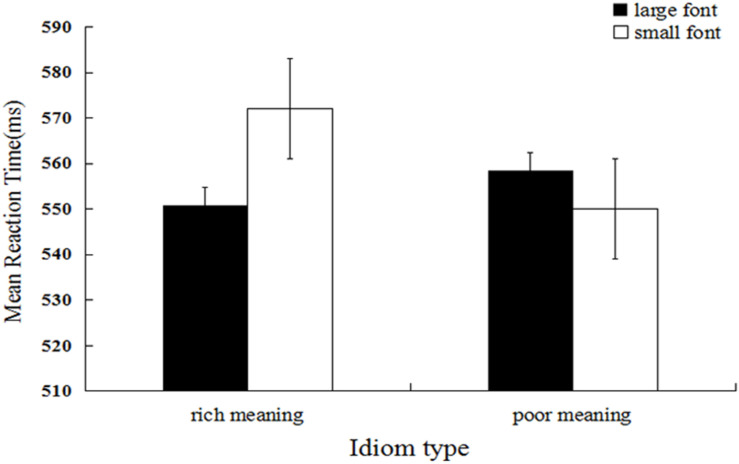
Mean reaction latency as a function of idiom type and idiom font size (Experiment 4).

Mean categorization latency was the dependent measure of interest^4^. We firstly performed log transformation of the data, and the untransformed means are reported. Response times that were outside 3 *SD*s from the mean (44 trials, 1.22%) and trials during which errors were made (261 trials, 7.25%) were excluded from the analysis (305 trials, 8.47%). A 2 (idiom type) × 2 (font size) repeated measures ANOVA was performed. The results showed that the main effect of idiom type was significant, and the mean reaction time for high-income occupations (*M* = 561.33, *SD* = 97.33) was significantly shorter relative to that for low-income occupations (*M* = 554.24, *SD* = 99.15), *F*(1, 29) = 6.14, *p* < 0.013, η*_p_*^2^ = 0.008. The main effect of font size was non-significant, *F*(1, 29) = 2.81, *p* = 0.094, η*_p_*^2^ = 0.004. As expected, the interaction between idiom type and font size was significant, *F*(1, 29) = 28.49, *p* < 0.001, η*_p_*^2^ = 0.037 (see Figure 4). Further simple effect analysis showed that the mean response time for idioms describing wealth in large font (*M* = 550.85, *SD* = 96.60) was significantly shorter relative to that for those in small font (*M* = 572.05, *SD* = 96.97), *F*(1, 29) = 23.63, *p* < 0.001, η*_p_*^2^ = 0.031. In contrast, the mean response time for idioms describing poverty in large font (*M* = 558.53, *SD* = 96.57) was significantly longer relative to that for those in small font (*M* = 550.01, *SD* = 101.50), *F*(1, 29) = 5.70, *p* = 0.017, η*_p_*^2^ = 0.008.

The results of Experiment 4 replicated the patterns in Experiments 1–3 and indicated that processing of idioms describing wealth and poverty also involved perceptual simulation of spatial size.

## General Discussion

In these four experiments, the representation of the important social categories wealth and poverty was grounded in spatial size simulation. Experiment 1 showed that responses to high-income occupations presented in large font were significantly faster relative to those to high-income occupations presented in small font. In contrast, responses to low-income occupations presented in large font were significantly slower relative to those to low-income occupations presented in small font. In Experiments 2, 3, and 4, we compared high-income occupations without social power and low-income occupations, names designated as those of rich and poor people, and abstract idioms describing wealth and poverty, respectively, and found similar results to those observed in Experiment 1. These results indicated that social categorization based on wealth involved perceptual simulation of spatial size.

The theory of grounded cognition proposes that conceptual thinking involves perceptual simulation, and cognizing abstract concepts can reactivate previously stored information from sensory–motor experience to form a simulation of this sensory–motor experience (Barsalou, 1999, 2008). Based on the perspective of embodied cognition, researchers have found that representation of social categories, such as different genders, involves simulation (Slepian et al., 2011, 2012; Zhang et al., 2014; Slepian, 2015). The results of the present study validate and support the theory of embodied cognition again, and extend prior research on social categories to wealth as well, which is another important social category.

Our results also provide new evidence indicating that early perceptual processes contribute to social categorization, which enriches the research on person construal (Cloutier et al., 2005; Macrae et al., 2005). Person construal research focuses on the effect of low-level perceptual processes on the activation of social categories and stereotypes (Cloutier et al., 2005; Macrae et al., 2005; Freeman and Ambady, 2011). In line with these research trends, the present study examined the effect of spatial size on social categorization based on the wealthy and found that spatial size processing can affect social categorization based on the wealthy. Further research should be conducted to explore the cognition and neural mechanism of how spatial size affects social categorization of the rich and the poor using event-related potential (ERP) and functional magnetic resonance imaging (fMRI) technology.

Barsalou (1999) proposed that perceptual experiences were derived from multiple sources of direct experience, and perceptual symbols were developed through schematization of daily experience. Categorization of individuals according to wealth is a very important and common dimension of social categorization in daily life (Leahy, 1981; Ramsey, 1991; Harvey and Bourhis, 2013). One of the largest differences between the rich and the poor, which can be schematized as perceptual experience, could be represented by a difference in spatial size in traditional Chinese culture. For example, in everyday life, relative to poverty, wealth is associated with larger houses and vehicles. These perceptual experiences could facilitate the construction of representations of rich and poor people; therefore, the process of social categorization based on wealth could be influenced by perceptual experience of spatial size. In addition, experiences in childhood play an important role in the formation of perceptual experience. Previous research has shown that children aged 3–11 years were able to classify people as poor or rich and believed that they differed with respect to certain traits such as intelligence (Leahy, 1981; Ramsey, 1991). In their everyday lives, children often observe the difference between wealth and poverty according to the dimension of spatial size; for instance, rich people choose larger houses and cars. These experiences could constitute the association between social categories and perceptual symbols (i.e., rich = large spatial size, poor = small spatial size).

Previous research has shown that spatial size metaphors affect power judgment (Schubert, 2005; Schubert et al., 2009), however, the current findings differed from those in such studies. For example, in Schubert et al. (2009) study, the task undertaken by participants involved power judgment, but in the current study, the task involved categorization according to wealth. In addition, social power and wealth are not equivalent concepts, in that wealthy people do not necessarily possess social power, and individuals with power are not necessarily wealthy. More importantly, in Experiment 2, we found that the categorization of rich people without social power (such as high school teachers) and poor people was affected by spatial size simulation. Therefore, the current results did not appear to be exclusively driven by social power. Future research based on experimental designs that clarify the possible role of social power in our conclusions is required.

Social categorization is a fundamental social-cognitive process, and categorization based on wealth is an important dimension of person perception (Leahy, 1981; Harvey and Bourhis, 2013). Given the important and foundational role of social categorization in the process of social cognition, including stereotyping and discrimination (Zhang et al., 2018), the current results have implications for numerous phenomena. For instance, they raise the possibility that simulation of different spatial sizes could provide a foundation for the representation of the social category of wealth. Therefore, further studies could clarify whether simulation of different spatial sizes influences the stereotyping of people as poor and rich. A previous study showed that larger spatial size was associated with positivity, and small spatial size was associated with negativity (Meier et al., 2008). We can infer that because social categorization of rich (vs. poor) people involves perceptual simulation associated with large (vs. small) spatial size, which is relevant to positive (vs. negative) valance, the perceptual simulation involving social categorization could play an important role in the formation of negative stereotypes toward poor people.

Several limitations of this study should be considered along with the results. First, in Experiments 1 and 2, we used high-income occupations and low-income occupations to represent the rich and the poor, which may indicate other information. Thus, future studies should verify the results of the current study using other more direct forms of representing the rich and the poor, such as pictures of the rich and the poor. Second, we used only Chinese participants in the present study and found that spatial size can affect social categorization based on wealth. Future studies should use participants from other cultural backgrounds (such as from Western cultures) to validate this effect. Finally, in the present study, we confirmed the effect of only spatial size on the processing of the rich and the poor. Further research should also be conducted to explore whether social categorization of the rich and the poor can affect processing of spatial size in order to systematically confirm the association between social categorization based on the wealthy and spatial size.

Overall, the present study demonstrates that spatial size can affect the social categorization of the rich and the poor and that social categorization based on wealth involves perceptual simulation of spatial size.

## Data Availability Statement

All datasets generated for this study are included in the article/Supplementary Material.

## Ethics Statement

The studies involving human participants were reviewed and approved by the Northwest Normal University, School of Psychology Ethics Committee. The patients/participants provided their written informed consent to participate in this study.

## Author Contributions

XZ conceived and designed the experiments. ZZ performed the experiments and analyzed the data. XZ and ZZ wrote the manuscript. Both authors contributed to the article and approved the submitted version.

## Conflict of Interest

The authors declare that the research was conducted in the absence of any commercial or financial relationships that could be construed as a potential conflict of interest.

## Footnotes

1 A 2 (occupation type) × 2 (font size) repeated measures ANOVA was performed to examine the participants’ error rates. The main effect of occupation type was significant, and the error rate for high-income occupations (*M* = 0.04, *SD* = 0.05) was significantly lower relative to that of low-income occupations (*M* = 0.10, *SD* = 0.10), *F*(1, 29) = 22.36, *p* < 0.001, η*_p_*^2^ = 0.44. The main effect of font size was non-significant, *F*(1, 29) = 3.53, *p* = 0.070, η*_p_*^2^ = 0.11. The interaction between occupation type and font size was significant, *F*(1, 29) = 8.34, *p* = 0.007, η*_p_*^2^ = 0.22. Simple effect analysis showed that the error rate for high-income occupations in large font size (*M* = 0.03, *SD* = 0.04) was lower relative to that for those in small font size (*M* = 0.05, *SD* = 0.06), and the difference was highly significant, *F*(1, 29) = 2.66, *p* = 0.11, η*_p_*^2^ = 0.08. In contrast, the error rate for large font size for low-income occupations (*M* = 0.13, *SD* = 0.12) was significantly higher relative to that for small font size (*M* = 0.08, *SD* = 0.07), *F*(1, 29) = 8.95, *p* = 0.006, η*_p_*^2^ = 0.24.

2 A 2 (occupation type) × 2 (font size) repeated measures ANOVA was performed to examine the participants’ error rates. The main effects of occupation type, *F*(1, 29) = 0.17, *p* = 0.683, η*_p_*^2^ = 0.006, and font size, *F*(1, 29) = 0.59, *p* = 0.447, η*_p_*^2^ = 0.020, were non-significant. The interaction between occupation type and font size was significant, *F*(1, 29) = 14.25, *p* = 0.001, η*_p_*^2^ = 0.329. Simple effect analysis showed that the error rate for high-income occupations in large font size (*M* = 0.11, *SD* = 0.16) was lower relative to that for those in small font size (*M* = 0.16, *SD* = 0.17), and the difference was highly significant, *F*(1, 29) = 13.75, *p* = 0.001, η*_p_*^2^ = 0.322. In contrast, the error rate for large font size for low-income occupations (*M* = 0.14, *SD* = 0.10) was significantly higher relative to that for small font size (*M* = 0.11, *SD* = 0.78), *F*(1, 29) = 5.75, *p* = 0.023, η*_p_*^2^ = 0.165.

3 A 2 (name type) × 2 (font size) repeated measures ANOVA was performed to examine error rates. The main effects of name type, *F*(1, 29) = 0.31, *p* = 0.58, η*_p_*^2^ = 0.11, and font size, *F*(1, 29) = 0.21, *p* = 0.65, η*_p_*^2^ = 0.007, were non-significant. The interaction between name type and font size was significant, *F*(1, 29) = 7.98, *p* = 0.008, η*_p_*^2^ = 0.22. Further simple effect analysis showed that the mean error rate for rich people’s names in large font (*M* = 0.03; *SD* = 0.05) was significantly lower relative to that for small font (*M* = 0.05; *SD* = 0.07), *F*(1, 29) = 4.14, *p* = 0.05, η*_p_*^2^ = 0.13. The mean error rate for poor people’s names in large font (*M* = 0.04, *SD* = 0.05) was higher relative to that for small font (*M* = 0.03, *SD* = 0.03), and the difference was significant, *F*(1, 29) = 2.81, *p* = 0.10, η*_p_*^2^ = 0.09.

4 A 2 (idiom type) × 2 (font size) repeated measures ANOVA was also performed to examine error rate. It was found that the main effect of idiom type was not significant, *F*(1, 29) = 0.45, *p* = 0.51, η*_p_*^2^ = 0.02. The main effect of font size was not significant, *F*(1, 29) = 0.85, *p* = 0.36, η*_p_*^2^ = 0.03. The interaction between idiom type and font size was not significant, *F*(1, 29) = 2.36, *p* = 0.14, η*_p_*^2^ = 0.08.
