# Supplementary material for: Spatial Size Can Affect Social Categorization of the Rich and the Poor
Source: Front Psychol. 2020 Aug 11;11:1914. doi: 10.3389/fpsyg.2020.01914 (PMC7432255; doi:10.3389/fpsyg.2020.01914)
Supplement: Supplementary file 2 [file Data_Sheet_2.docx]

**Appendix**

Experiment 1

**low-income occupations:**

农民- peasant

菜农-vegetable grower

保安- security

汽修工-motor mechanic

小商贩-petty dealer

装卸工-loader

环卫工-sanitation worker

打字员-typist

油漆工-painter

服务员-waiter

清洁工-cleaner

快递员- delivery man

建筑工人-building worker

商场销售-salesman

饭店招待-hotel waiter

公交司机-bus driver

**high-income occupations：**

总统-president

总裁-CEO

省长-provincial governor

大法官-justice

董事长-chairman

理事长-president of a council

检察官-procurator

总司令-commander in chief

执行官-executive officer

国务卿-secretary of state

行政官-administrator

总经理-general manager

银行行长-bank director

税务局长-director of tax office

财务部长-financial minister

国防部长-defense minister

Experiment 2

**low-income occupations:**

农民- peasant

菜农-vegetable grower

保安- security

汽修工-motor mechanic

小商贩-petty dealer

装卸工-loader

环卫工-sanitation worker

打字员-typist

油漆工-painter

服务员-waiter

清洁工-cleaner

快递员- delivery man

建筑工人-building worker

商场销售-salesman

饭店招待-hotel waiter

公交司机-bus driver

**high-income occupations：**

教授-professor

医生-doctor

演员-actor

播音员-announcer

设计师-stylist

科学家-scientist

咨询师-counselor

工程师-engineer

运动员-athlete

企业家-entrepreneur

主持人-compere

程序员-programmer

大学教师-university teacher

科研人员-researcher

银行职员-bank staff

空中小姐-air hostess

Experiment 3

**names**

赵楚妙 孙建国 李丽娟 周永亮 吴兰凤 郑德忠

张晓军 刘秀莹 陈伟强 杨淑芳 郭建州 徐兰芳

胡洁珊 孙国仁 高巧芬 朱明强 何玉珍 林俊伟

马建军 罗娇芸 梁子豪 宋瑞莺 郑有国 谢彩霞

唐艳丽 冯晓鹏 王丽琴 赵林涛 韩文浩 郭佳媚

Experiment 4

**rich-meaning idiom**：

腰缠万贯- wallow in money

日富月昌-become rich and prosperous day by day

有钱有势-rich and influential

家给人足- houses have adequate supplies and people live in contentment.

一掷千金- spend gold on one throw

日进斗金-produce shovel-loads of cash

珠光宝气-bedecked with jewels

富商大贾-wealthy trader

万贯家私-as extremely wealthy as a millionaire

朱门绣户-rich and powerful family

富有四海-possess all within the whole country

铜山金穴- copper mountain and gold cave

堆金积玉-heap up gold and accumulate jade

养尊处优-enjoy in affluence

钟鸣鼎食-living an extravagant life

**poor-meaning idiom：**

身无分文-not having a single penny left on

一贫如洗-be desperately poor

衣衫褴褛-be shabby in dress

饥寒交迫-suffer from hunger and cold

一无所有-have nothing at all

缺衣少食-not having enough food and clothing

食不果腹-have little food to eat

节衣缩食-tighten one’s belt

忍饥挨饿-suffer from starvation

囊空如洗-penniless

一穷二白-be poor and blank

地瘠民贫-desert land and poor people

衣食单薄-thin food and clothing

破衣烂衫-ragged clothes

家徒四壁-be utterly destitute
